# “Affimer” synthetic protein scaffolds block oxidized LDL binding to the LOX-1 scavenger receptor and inhibit ERK1/2 activation

**DOI:** 10.1016/j.jbc.2023.105325

**Published:** 2023-10-05

**Authors:** Barnaby W.R. Roper, Christian Tiede, Izma Abdul-Zani, Gary A. Cuthbert, Dhananjay Jade, Ahmed Al-Aufi, William R. Critchley, Queen Saikia, Shervanthi Homer-Vanniasinkam, Tatsuya Sawamura, Michael J. McPherson, Michael A. Harrison, Darren C. Tomlinson, Sreenivasan Ponnambalam

**Affiliations:** 1 School of Molecular & Cellular Biology, University of Leeds, Leeds, UK; 2 Leeds Vascular Institute, Leeds General Infirmary, Leeds, UK; 3 School of Biomedical Sciences, University of Leeds, Leeds, UK; 4 Department of Physiology, Shinshu University, Nagano, Japan

**Keywords:** LOX-1, scavenger receptor, oxidized low-density lipoprotein, Affimer, MAPK signaling, lectin-like domain

## Abstract

In multicellular organisms, a variety of lipid-protein particles control the systemic flow of triacylglycerides, cholesterol, and fatty acids between cells in different tissues. The chemical modification by oxidation of these particles can trigger pathological responses, mediated by a group of membrane proteins termed scavenger receptors. The lectin-like oxidized low-density lipoprotein (LOX-1) scavenger receptor binds to oxidized low-density lipoprotein (oxLDL) and mediates both signaling and trafficking outcomes. Here, we identified five synthetic proteins termed Affimers from a phage display library, each capable of binding recombinant LOX-1 extracellular (oxLDL-binding) domain with high specificity. These Affimers, based on a phytocystatin scaffold with loop regions of variable sequence, were able to bind to the plasma membrane of HEK293T cells exclusively when human LOX-1 was expressed. Binding and uptake of fluorescently labeled oxLDL by the LOX-1-expressing cell model was inhibited with subnanomolar potency by all 5 Affimers. ERK1/2 activation, stimulated by oxLDL binding to LOX-1, was also significantly inhibited (*p* < 0.01) by preincubation with LOX-1-specific Affimers, but these Affimers had no direct agonistic effect. Molecular modeling indicated that the LOX-1-specific Affimers bound predominantly *via* their variable loop regions to the surface of the LOX-1 lectin-like domain that contains a distinctive arrangement of arginine residues previously implicated in oxLDL binding, involving interactions with both subunits of the native, stable scavenger receptor homodimer. These data provide a new class of synthetic tools to probe and potentially modulate the oxLDL/LOX-1 interaction that plays an important role in vascular disease.

To allow systemic circulation between the hepatic and peripheral tissues, water-insoluble lipid molecules such as triacylglycerides, cholesterol, and cholesterol esters are packaged with specific apoproteins to generate amphipathic lipoprotein complexes. In particular, the action of lipases on very low-density lipoprotein complexes, synthesized in the liver in order to move endogenous lipids to extrahepatic tissues, gives rise to low density lipoprotein (LDL). This complex, containing a high proportion of cholesterol esters within a hydrophobic core surrounded by a phospholipid monolayer and apoprotein B-100, is associated with high circulating cholesterol and is a prognostic indicator for cardiovascular disease (1). LDL accumulated in the intima of arterial blood vessels is subject to chemical modification and oxidation by free radicals such as reactive oxygen species and hydroxyl ions released by actively respiring endothelial and vascular smooth muscle cells and as a consequence of mechanical shear stress. Oxidation of both lipidic (2, 3, 4) and protein components (3, 5, 6) may occur. Interaction of oxidized LDL (oxLDL) with specific endothelial cell receptors leads to monocyte chemoattractant protein 1 and macrophage colony-stimulating factor release that initiates monocyte recruitment and invasion and differentiation into macrophages (7). Uptake of oxLDL by macrophages is the first stage in the formation of foam cells and consequent atherosclerotic plaque formation. Accumulation of LDL also initiates signaling processes (8) that trigger an inflammatory response that contributes to vascular dysfunction associated with a range of pathologies (9).

oxLDL uptake and accumulation in endothelial cells and macrophages is facilitated by scavenger receptors, a diverse group of membrane proteins that recognize a wide range of proteins, lipids, lipid particles, and pathogens (10, 11). The Class E scavenger receptor, lectin-like oxidized low-density lipoprotein receptor (LOX-1, SR-E1, and OLR1) is a type II membrane protein with a short cytoplasmic N-terminal region, single transmembrane segment followed by a helical “neck” domain (residues 61–136) that precedes a C-type lectin-like fold domain (residues 136–273) (12, 13, 14). LOX-1 homodimers linked by disulphide bonds promote oxLDL recognition, but although LOX-1 mutational studies have implicated a series of positively charged residues within the lectin-like domain, the exact nature of LOX-1/oxLDL interaction remains uncertain (15, 16, 17). LOX-1 is normally expressed at relatively low levels in most cells, but is upregulated in response to proinflammatory stimuli (such as reactive oxygen species or glucose) and shear stress (18). Allelic polymorphisms within the human *OLR1* locus that encodes LOX-1 are associated with altered risk of cardiovascular disease (19) and stroke (20, 21). Studies on genetically engineered mouse strains that display increased atherosclerosis in coronary arteries suggest that LOX-1 promotes disease, that is, is a proatherogenic factor (22, 23). This has led to much interest in whether targeting LOX-1 could be beneficial for alleviating or reducing atherosclerosis and arterial disease (19, 24).

Antibodies have long been established as biological agents that can be engineered to bind specific targets both as analytical tools and as therapeutics. While not yet approved for clinical use in the treatment of atherosclerosis, antibodies directed against oxLDL block proinflammatory signaling in macrophages *via* modulation of the p38/mitogen activated protein kinase (MAPK) and NF-kB pathways (25). Antibody-based applications can however present problems: the requirement for animal or animal cell expression, stability and batch variability can impact on their usefulness and cost-effectiveness. Monoclonal antibody therapeutics in particular are extremely costly. A number of technologies have been developed within the last decade that aim to incorporate the intrinsic specificity of antibodies into small, highly stable, and easy to produce synthetic protein scaffolds suitable for use as highly selective biomolecular probes or biopharmaceuticals (26). These synthetic proteins, such as Adnectins (27), Designed Ankyrin Repeat Proteins (DARPins) (28, 29) and Affimers (30, 31). These synthetic proteins share a common set of design principles that counter some of the issues relating to antibodies. Comparatively, synthetic scaffolds are smaller, have a simple stable fold and require no posttranslational modifications, allowing them to be produced in rapidly and cost effectively in bacterial expression systems. Cytoplasmic expression can also facilitate analysis of intracellular signaling pathways, for example.

Affimers are novel antigen-binding reagents whose structure is based on a consensus of 57 plant phytocystatin sequences that gives the structure a high level of stability (T_m_ ∼101 °C). This phytocystatin scaffold lacks disulphide bonds (30). The core protein scaffold comprises a single α-helix supported by a β-sheet formed of four antiparallel ß-strands (Fig. 1*D*). Phytocystatins are cysteine proteases and have two inhibitory loops which are replaced in the Affimer by variable nine-residue flexible loops variable region (VR)1 and VR2 ((30), Fig. 1*D*). The sequence of these binding loops has been randomized in the creation of a phage display library containing 1.3 × 10^10^ independent clones (30), each phage engineered such that their capsid coat proteins display the randomized VR1 and VR2 for binding target antigens, which can include proteins, lipids, DNA, or small molecules. Iterative phage display-panning under increasing stringency leads to isolation of Affimers exhibiting specific binding to a chosen target (Fig. 1*A*). Phage isolated from rounds of panning are amplified, and final phage clones are used to clone Affimer sequence(s) for bacterial expression. This approach has been used for example to identify Affimers capable of binding endothelial vascular endothelial growth factor R2 and inhibit vascular endothelial growth factor-A-stimulated signal transduction and hence block endothelial tubulogenesis (30).

**Figure 1:**
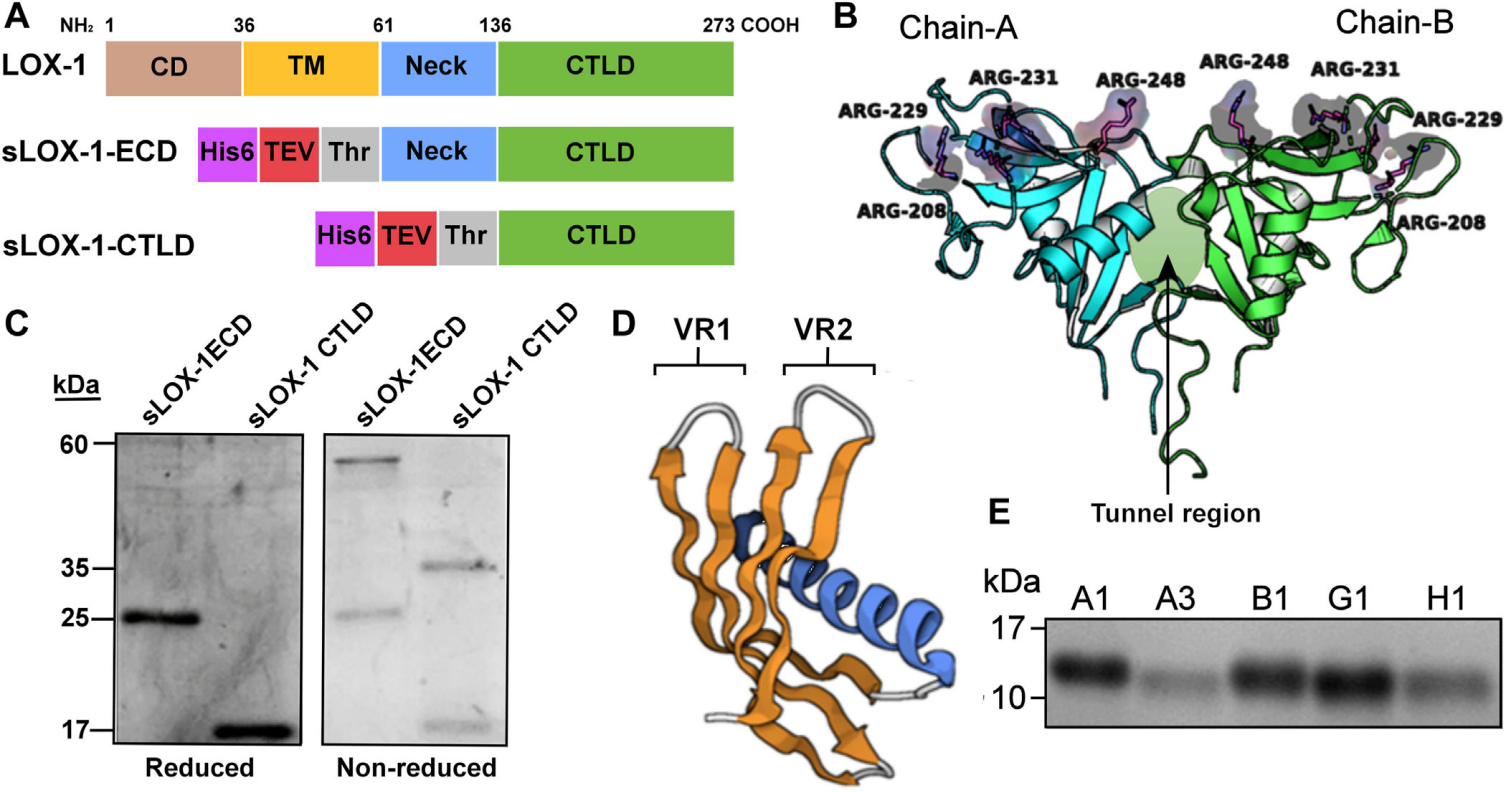
**Affimer proteins recognize recombinant soluble lectin-like domain of scavenger receptor LOX-1.***A*, schematic for the recombinant LOX-1 soluble domain used as targets for Affimer identification. CD, C-terminal (intracellular domain); CTLD, C-terminal lectin-like domain; His6, polyhistidine affinity tag; Neck, neck domain linking the lectin-like domain and transmembrane helical segment; TEV, TEV protease cleavage site; TM, transmembrane segment; Thr, thrombin cleavage site. *B*, structural model of dimeric LOX-1 extracellular domain (PDB code 6Tl9). The Arg residues on chains A (*cyan*) and B (*green*) are implicated in Apoprotein B100 binding. The “Tunnel” region indicated at the dimer interface is implicated in lipid and hydrophobic drug binding. *C*, SDS-PAGE analysis of recombinant LOX-1 soluble domains (as in *B*) under reducing (*left*) and nonreducing (*right*) conditions. *D*, 3-D structural representation of the Affimer backbone showing the four-strand anti-parallel β-sheet (*yellow*) and single α-helix (*blue*). Loop regions showing extensive sequence variability within the Affimer library are shown in *white* (VR1, VR2). *E*, SDS-PAGE analysis of the purified Affimers identified as binding to sLOX-1. CTLD, C-terminal lectin-like domain; PDB, Protein Data Bank; TEV, tobacco etch virus; VR, variable region.

Monoclonal antibodies to LOX-1 administered to both cell and animal models have been shown to effect a reduction in pathophysiology consistent with a block in LOX-1-mediated proatherogenic effects (32, 33, 34). More recently, a number of different approaches have been used to identify LOX-1-specific agents including small chemical molecules (35, 36), peptides (37), polyinosinic acid, and carrageenan (38). Hence, it is clear that there is potential for engineered molecules to have the capacity to modulate LOX-1/oxLDL interaction, with the potential for antiatherogenic effects or to be useful as probes to study the interaction. The capacity of Affimers to block membrane protein-ligand interactions with consequences for signaling and phenotypic modulation (30) suggest their suitability for this purpose. This study aimed to identify a range of Affimers capable of highly specific interaction with the LOX-1 receptor and exerting inhibitory effects on both oxLDL uptake into cells and intracellular signaling mediated by the receptor. Such molecules have the potential to provide new therapeutic strategies that block the proatherogenic effects of LOX-1.

## Results

### Screening, expression, and characterization of LOX-1-specific affimers

Previous studies have demonstrated that Affimer synthetic protein scaffolds can recognize a variety of molecules based on electrostatic noncovalent interactions (30, 31). In this study, recombinant forms of the extracellular region of LOX-1 were produced in *Escherichia coli* in the form of inclusion bodies that could be dissolved and subsequently refolded (Fig. 1*C*), confirmed by circular dichroism spectroscopy (data not shown). When analyzed by SDS-PAGE under reducing conditions, the polyhistidine-tagged LOX-1 extracellular domain has a molecular mass of 25 kDa, whereas the C-terminal lectin-like domain (CTLD) has a mass of 17 kDa (Fig. 1*C*, left panel). Both proteins are capable of forming discrete dimeric species linked by intermolecular disulphide bonds under nonreducing conditions (Fig. 1*C*, right panel), consistent with successful adoption of an authentic quaternary structure. Using both forms of homodimeric scavenger receptor extracellular domain as targets, iterative panning of the Affimer bacteriophage library identified five molecules as potential specific binding partners for LOX-1. These Affimer candidates, designated A1, A3, B1, G1, and H1, were subsequently purified from an *E. coli* T7-based expression system with high yield (>10 mg/L culture) and to high levels of purity (>99% by SDS-PAGE staining) for direct testing for binding to recombinant human and mouse LOX-1 (Fig. 1*E*).

**Figure 2:**
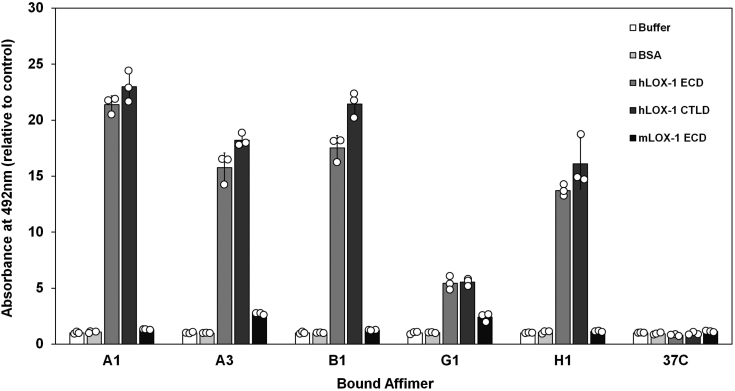
**Affimers associate with recombinant soluble LOX-1 receptor.** ELISA assays of Affimer binding to recombinant human LOX-1 extracellular domain (hLOX-1 ECD: *mid-gray*), C-terminal lectin-like domain (hLOX-1 CTLD: *dark gray*) or recombinant mouse LOX-1 extracellular domain (mLOX-1 ECD: *black*). Affimer 37C is a negative control previously characterized as specifically binding to human vascular endothelial growth factor receptor. Buffer controls for background signal and BSA controls for nonspecific binding to the ELISA plate are also shown. Bars indicate mean values, error bars indicate SD. Individual data points for n = 3 assays are shown (*white circles*). BSA, bovine serum albumin; CTLD, C-terminal lectin-like domain; ECD, extracellular domain.

The five candidate LOX-1-specific Affimers were expressed and purified and subsequently directly tested for binding to immobilized human and mouse soluble LOX-1 proteins (Fig. 2). All five candidates gave strong signals compared to buffer, nonspecific binding controls (bovine serum albumin [BSA] and negative control, Affimer 37C), consistent with highly specific binding to the soluble LOX-1 proteins. Affimer 37C, BSA, and buffer controls all showed similar negligible readouts. This indicates consistent, stable baseline values with minimal nonspecific binding against which it is possible to quantify LOX-1-specific recognition of Affimers. There were no significant differences (*p* > 0.01) in binding between the Affimers and either LOX-1 CTLD or extracellular domain (ECD) proteins, consistent with all five Affimers binding to the CTLD without involvement of the “neck” domain. Affimer G1 displayed the lowest apparent binding to either sLOX-1-ECD or sLOX-1-CTLD; however, this was >3-fold greater than baseline and negative control values (Fig. 2). Only Affimers A3 and G1 also exhibited appreciable binding to recombinant mouse sLOX-1 compared to negative controls. Human and mouse LOX-1 ECDs share 61% overall identity at the primary structure level, with a higher level of conservation (73% identity) specifically among residues in the 229 to 262 region that contribute to the putative oxLDL binding surface.

### Affimers block LOX-1-mediated binding of oxLDL to the cell surface

**Figure 3:**
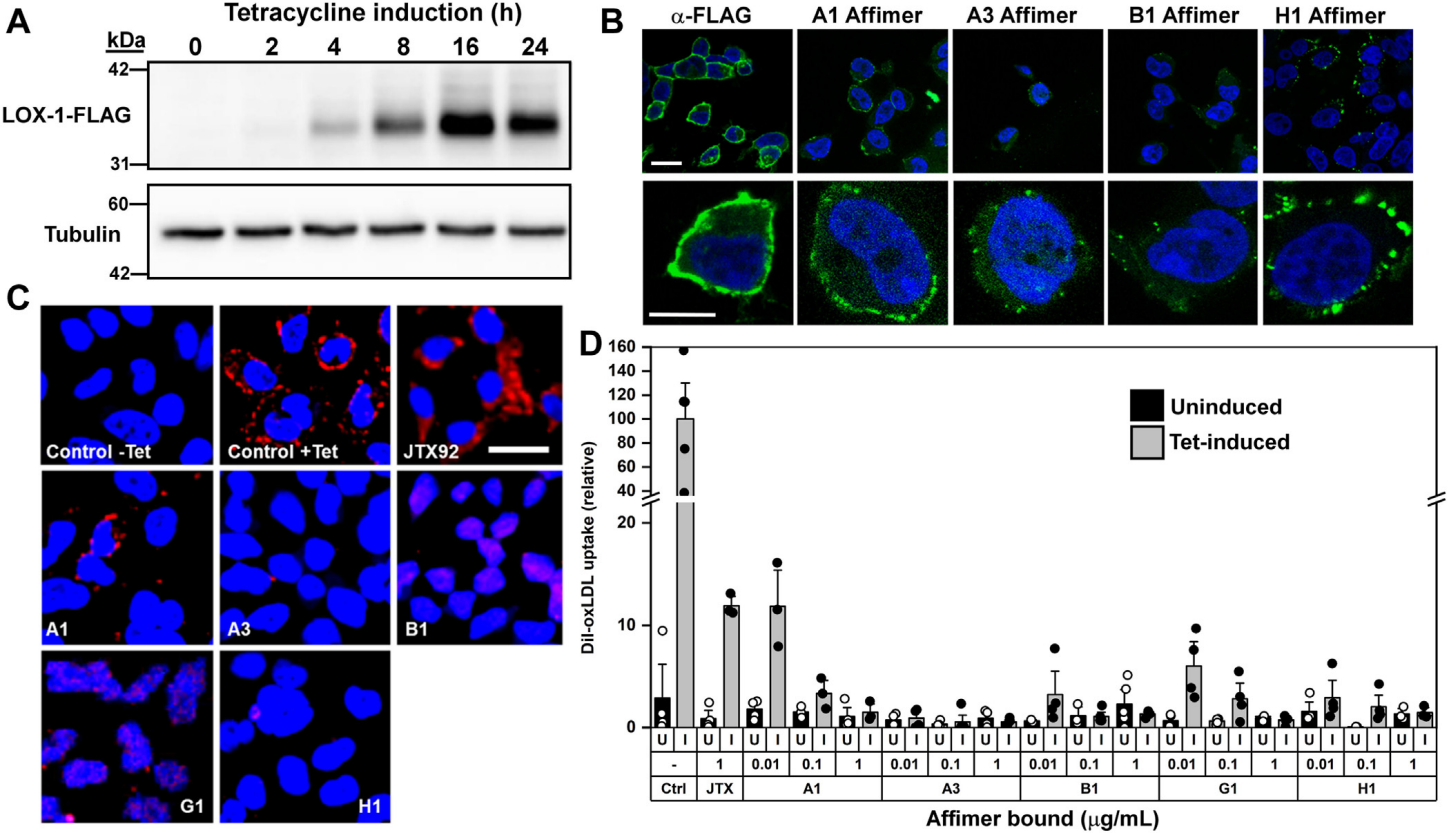
**Affimers inhibit oxLDL binding to cell surface LOX-1-FLAG in HEK293 cells.***A*, tetracycline induces expression of human LOX-1 in HEK293T “Tet-On” cells. Whole cell lysates (separated by SDS-PAGE, 10 μg protein per well) were probed by immunoblotting with anti-FLAG monoclonal antibody after 24 h induction with tetracycline. *B*, fixed, nonpermeabilized HEK293-LOX-1-FLAG cells show surface labeling with AlexaFluor 488 maleimide–labeled Affimers (*green*). Bottom row shows expanded view of individual cells to illustrate the Affimer staining patterns. Anti-FLAG MAb binds to the extracellular-exposed FLAG tag on LOX-1. Scale bar indicates 20 μm. *C*, binding and uptake of Dil-labeled oxLDL (*red*) requires tetracycline-induced LOX-1 expression and is inhibited by Affimers. In these representative images, signal indicating bound/internalized oxLDL is seen in control induced cells (Control +Tet) but not in the uninduced control (Control-Tet). oxLDL signal decreases when cells are preincubated with Affimers or with the anti-LOX-1 antibody JTX92. Microscopy scale bar is 50 μm. Nuclei are stained with DAPI. *D*, quantitation of the effects of Affimers on Dil-labeled oxLDL binding. LOX-1-FLAG expression was induced with tetracycline (I: *gray bars*) or remained uninduced (U: *black bars*) as a negative control. HEK293-LOX-1-FLAG cells were preincubated with 0.01, 0.1, or 1 μg/ml Affimer. Blocking antibody JTX92 was used at 1 μg/ml. Microscopy fields from at least three individual experiments were analyzed using ImageJ (see Experimental procedures) and the values expressed relative to the tetracycline-induced positive control containing no Affimer. Error bars indicate SD. Individual replicate values are shown as *white* or *black circles* for induced and uninduced cells, respectively. DAPI, 4′,6-diamidino-2-phenylindole; oxLDL, oxidized low-density lipoprotein.

Affimers proposed to be LOX-1-specific should also effectively compete with oxLDL for binding to the scavenger receptor in a cellular context. However, normal LOX-1 levels in most nonvascular cells and tissues are negligible, making commonly used cell lines unsuitable as models for studying oxLDL/LOX-1 interaction. To address this, a cell-based model was constructed that allows cell surface expression of human LOX-1. Given that increased LOX-1 expression in response to shear stress or reactive oxygen species can promote cellular apoptosis (39), control over expression in the cell model is essential. A HEK293T cell line was engineered to include tetracycline-inducible (Tet-On) expression of C-terminal FLAG-tagged human LOX-1 using Flp-In recombination technology (HEK293T-LOX-1-FLAG). Immunoblot analysis of whole cell lysates after tetracycline induction shows maximal LOX-1-FLAG expression ∼16 h after tetracycline induction (Fig. 3*A*). Immunofluorescence labeling of nonpermeabilised HEK293T-LOX-1-FLAG cells with anti-FLAG antibody showed distinctive surface labeling consistent with binding to extracellular epitopes of plasma membrane-bound proteins (Fig. 3*B*). Thus, in this cell line, LOX-1-FLAG is present at the plasma membrane and is accessible to antibodies directed to the epitope fused to the carboxyl terminus of the extracellular lectin-like domain of LOX-1. Direct labeling with AlexaFluor488–conjugated Affimers A1, A3, B1, and H1 also showed plasma membrane binding (Fig. 3*B*). However, there were qualitative differences in this labeling: Consistently, A1 and H1 showed the highest plasma membrane labeling, but whereas A1 staining was regular (similar to that seen with the anti-FLAG antibody), H1 staining was seen in intensely staining patches or subdomains. Affimers, A3 and B1, also showed irregular staining of plasma membrane subdomains or patches (Fig. 3*B*). Nonetheless, LOX-1-specific Affimers clearly show recognition of cellular LOX-1, with no detectable binding to uninduced cells (data not shown).

Previous studies have shown that oxLDL undergoes LOX-1-dependent cell surface binding and endocytosis (40, 41, 42). Binding of oxLDL, fluorescently labeled with the lipophilic dye Dil, to scavenger receptor on the surface of HEK293T-LOX-1-FLAG cells, was assessed by quantitative microscopy (Fig. 3, *C* and *D*). Noninduced cells showed minimal, nonsignificant binding of oxLDL (Fig. 3, *C* and *D*). Induction of LOX-1-FLAG by addition of tetracycline to the culture medium for 16 h caused a significant (*p* < 0.001) ∼40-fold increase in binding of oxLDL to the cell surface and evidence of uptake into the cells by endocytosis (Fig. 3*D*). The monoclonal antibody JTX92 is well-established as a tool for blocking oxLDL binding to human LOX-1 (33). Here, exposing HEK293T-LOX-1-FLAG cells to the JTX92 antibody (at 1 μg/ml, ∼6 nM) resulted in an ∼88.2 ± 0.6% decrease in oxLDL signal (Fig. 3*D*). In comparison, the panel of five LOX-1-specific Affimers all caused a concentration-dependent decrease in oxLDL signal in the cells even at subnanomolar concentrations (Fig. 3*D*). Affimer A1 showed 88.4 ±2.5% inhibitory effect at 0.01 μg/ml (equivalent to ∼0.74 nM for this 13.6 kDa polypeptide), increasing to >98% inhibition at 1 μg/ml (∼74 nM). Affimers A3, B1, G1, and H1 were even more effective, consistently achieving >90% inhibition at subnanomolar concentration and >98% inhibition at the maximum concentration tested (1 μg/ml, ∼74 nM) (Fig. 3*D*). It is clear from these data that all five identified Affimers are very potent inhibitors of oxLDL binding to the scavenger receptor LOX-1 in this engineered cell model.

### LOX-1-specific Affimer inhibition of oxLDL-stimulated signal transduction

**Figure 4:**
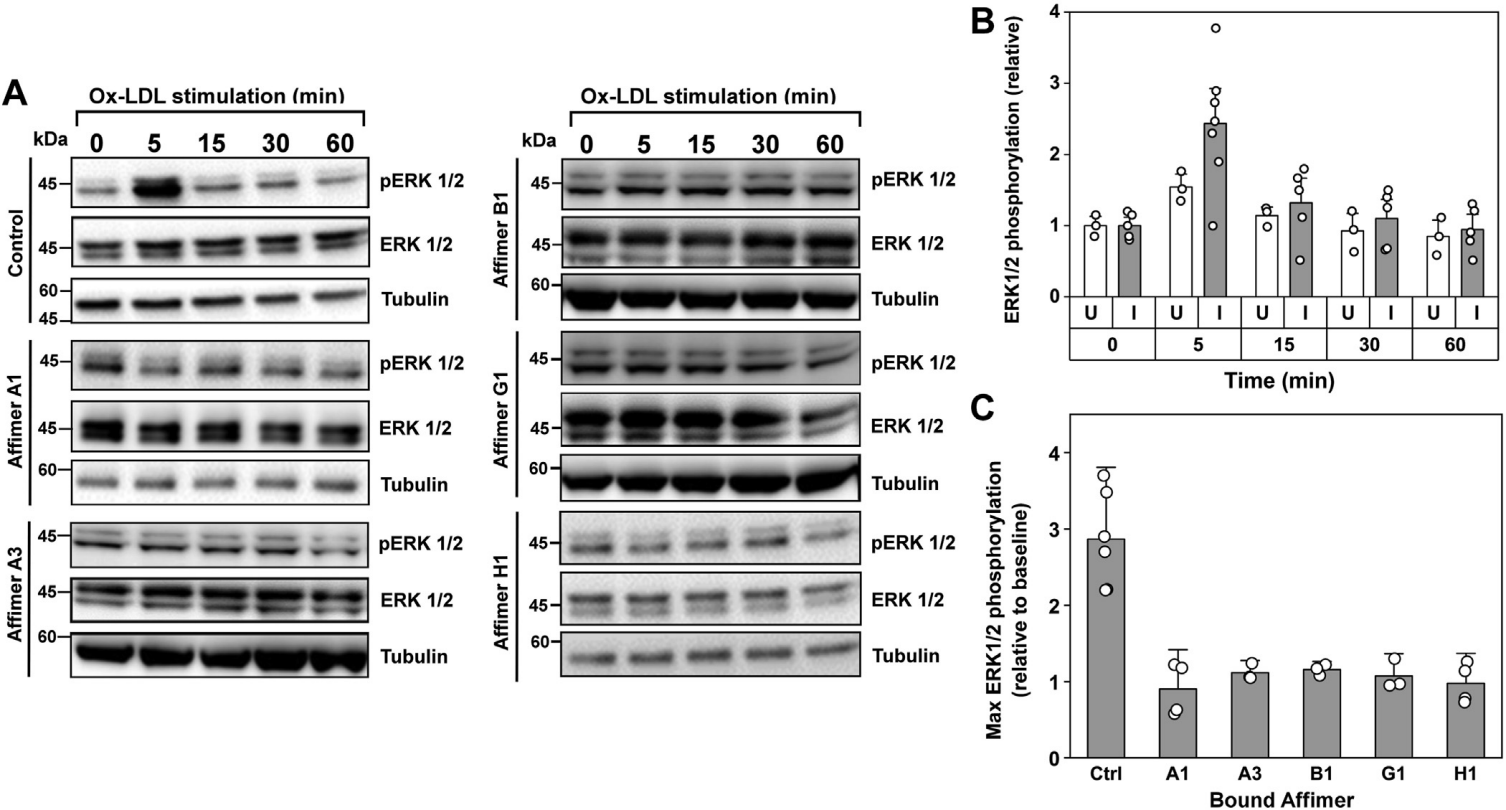
**Affimers inhibit LOX-1-mediated ERK1/2 activation.***A*, immunoblotting shows maximal ERK1/2 phosphorylation after 5 min exposure of HEK293-LOX-1-FLAG cells to oxLDL (100 μg/ml). Total ERK1/2 is unaffected. Affimers (100 ng/ml) abolish the peak in ERK1/2 phosphorylation. Signal for tubulin is used to ensure consistent loading of the lanes on the immunoblot. *B*, quantitation of the time course of oxLDL-dependent ERK1/2 phosphorylation in tetracycline-induced (*gray bars*) and uninduced (*white bars*) HEK293-LOX-1-FLAG cells. Values are means of 5 (induced) or 3 (uninduced) individual experiments, expressed relative to the time zero (oxLDL-independent) values. *C*, quantitation of Affimer inhibition of oxLDL/LOX-1-mediated activation of ERK1/2. Values are expressed relative to the corresponding basal, oxLDL-independent ERK1/2 phosphorylation at time zero for each time course. All Affimers were significantly inhibitory at the *p* < 0.05 level. Values are means of at least three individual experiments. Error bars indicate SD. Individual values in each dataset are shown by *white circles*. ERK, extracellular-regulated kinase; oxLDL, oxidized low-density lipoprotein.

**Supplemental Fig. S1:**
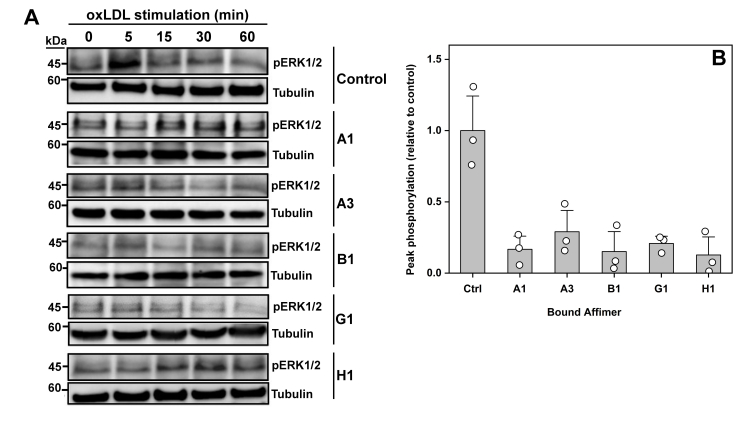
**Affimers show no direct agonistic effect on the LOX-1 scavenger receptor.***A*, immunoblot detection of ERK1/2 phosphorylation in whole cell lysates after incubation of LOX-1-FLAG HEK293 cells with Affimer protein (10 μg/mL) for the times indicated. As a positive control, cells were incubated with oxidized LDL (oxLDL; 10 μg/mL) leading to maximal ERK1/2 phosphorylation after 5 min (see also Fig. 5, main text). Immunoblot signal for tubulin is included as an indicator of equivalent sample loading. *B*, Quantitation of ERK1/2 phosphorylation in response to Affimer binding to HEK293 LOX-1-FLAG cells. Mean values (n = 3) are expressed relative to the maximal phosphorylation signal in the oxLDL-stimulated positive control (Ctrl). Individual replicate values are shown as white circles. Error bar indicates SD.

oxLDL is reported to stimulate intracellular signaling *via* extracellular-regulated kinase (ERK)1/2 activation (10). The inhibitory effect of Affimers on ERK1/2 activation by phosphorylation was evaluated in the LOX-1-expressing HEK293T cell model (Fig. 4). This assay, comparing oxLDL-dependent increases in immunoblot signal for ERK1/2 phosphorylated at Thr202/Tyr204, showed a peak of ERK1/2 phosphorylation after 5 min exposure to oxLDL (Fig. 4, *A* and *B*). ERK1/2 phosphorylation was significantly higher (*p* < 0.05) in cells induced to express LOX-1 than in uninduced cells. No significant difference in stimulation was observed whether oxLDL was applied at 10 or 100 μg/ml (data not shown). Thus, the HEK293-LOX-1-FLAG cells show a significant signaling reaction that was dependent on both LOX-1 expression and oxLDL binding. Subsequently, ERK1/2 phosphorylation rapidly diminished to basal levels, as previously described (10). Preincubation of HEK293-LOX-1-FLAG cells with any of the LOX-1-specific Affimers significantly inhibited (*p* < 0.05) oxLDL-dependent ERK1/2 activation (Fig. 4, *A* and *C*). There is a noticeable (but not statistically significant) effect of oxLDL on ERK1/2 phosphorylation even in uninduced HEK293-LOX-1-FLAG cells (Fig. 4*B*), perhaps due to low level leaking expression of LOX-1, or the presence of alternative scavenger receptors in these cells. No direct agonistic effect of Affimer binding to LOX-1 on ERK1/2 activation was detected (Fig. S1). However, from the data in Figure 4 it can be concluded that the identified Affimers are able to bind with a high degree of specificity to human LOX-1 and are potent inhibitors of both the oxLDL binding/uptake and oxLDL-mediated signaling functions of this scavenger receptor.

### Modeling of Affimer binding to LOX-1

**Figure 5:**
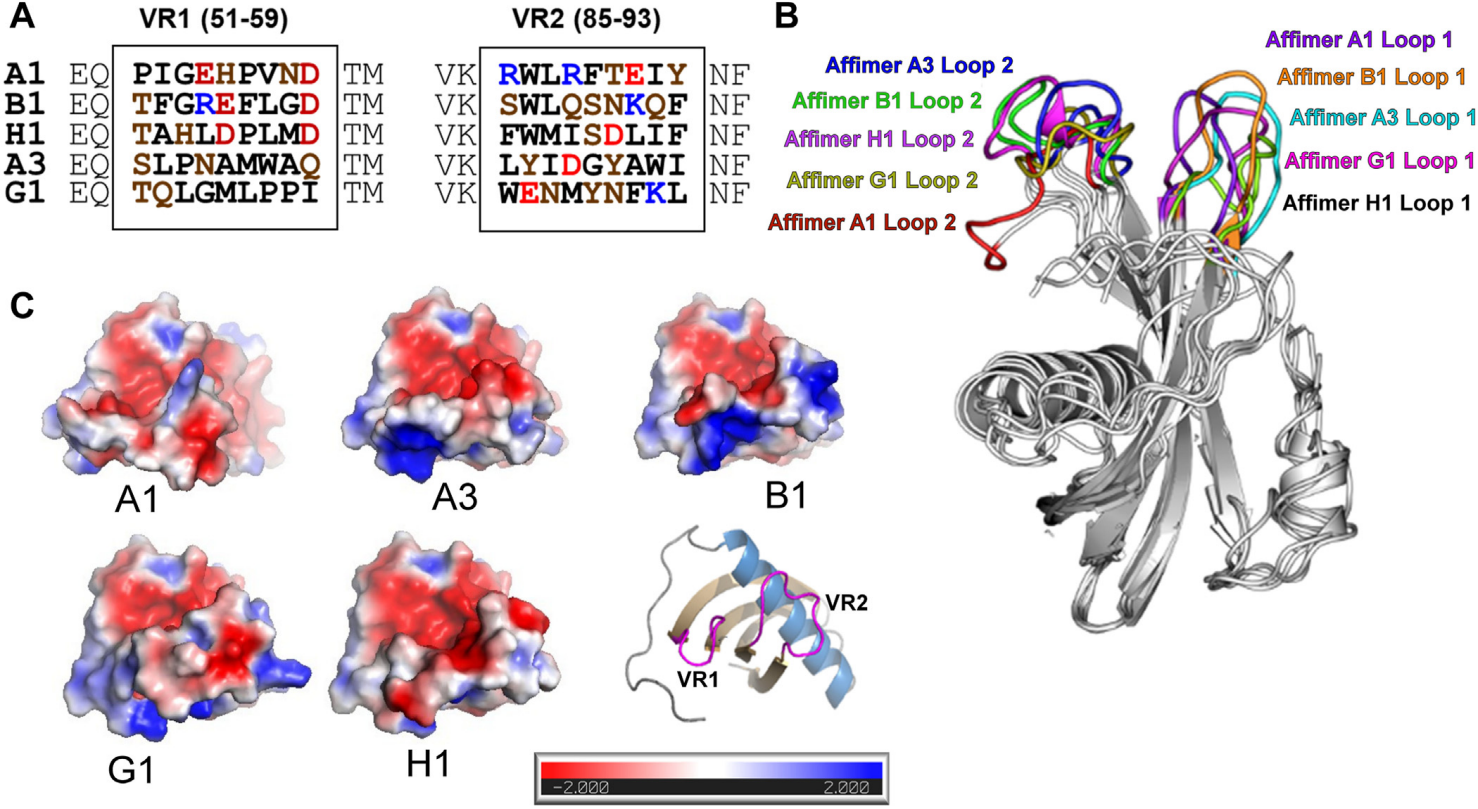
**Modeling of LOX-1-specific Affimers.***A*, amino acid sequences of the variable loop regions (VR1, VR2) of Affimers found to bind to recombinant LOX-1. Polar residues are *brown*, hydrophobic residues *black*, negatively charged residues *red* and positive *blue*. Numbers indicate the residue numbers within the full Affimer sequence. *B*, superimposed energy-minimized models of Affimers A1, A3, B1, G1, and H1 showing conformational variability within VR1 (Loop 1) and VR2 (Loop 2). *C*, surface electrostatics of the five LOX-1 specific Affimers (+2 to −2 scale). Affimer surfaces formed primarily by the VR loops are orientated toward the viewer, as shown by the cartoon representation bottom right. VR, variable region.

Amino acid sequences of the VR loops of the candidate LOX-1-binding Affimers were deduced from the corresponding nucleotide coding sequences of isolated plasmid, but showed only very limited sequence similarity within these nine residue segments (Fig. 5*A*). Affimers A1, B1, and H1 do each contain two negatively charged residues in similar positions within VR1, but similar resides are absent from A3 and G1. G1 in particular shows a higher proportion of hydrophobic residues in VR1. VR2 shows some broad similarities across all five Affimers for distribution of hydrophobic/aromatic residues, with this residue type in all Affimers at position 93 and in all but G1 at position 86 (Fig. 5*A*). Energy-minimized models of the five Affimers (Fig. 5*B*), selected based on the optimal C-Score (Table S1), show differences in conformation of the VR loops (Fig. 5*B*), which combined with the variability in sequence, gives rise to quite different and distinct electrostatics across the VR1/2 surfaces that can be presumed to be the principle LOX-1 binding regions (Fig. 5*C*). Clearly, the quite variable geometries of negative, positive, and nonpolar areas on the surface of the Affimers must still be complementary to the surface of LOX-1 to which they apparently bind. However, the extent of the differences between Affimers implies that the exact mode of binding should be different for each molecule. In addition, some conformational flexibility, induced for example by binding to the target LOX-1, cannot of course be excluded.

For modeling of interactions between the Affimers and soluble LOX-1 domain, a homodimeric structure was derived from the published crystallographic model (Fig. 1*B*). Chain-A of this model contains 129 residues (141–270) and chain-B contains 128 residues (141–269). The fold of the CTLD domain of LOX-1 comprises two α-helix segments and eight β-sheets in each monomer, with both chains contributing arginine residues to the surface of the CTLD domain (R208, R229, R231, and R248: Fig. 1*B*). These residues are implicated in the interaction with the Apoprotein B-100 component of oxLDL (15). In the mouse protein, R208 and R231 are conserved, and the positive charge of R229 is conserved as a lysine residue. The “Tunnel region”, also proposed as a site involved in lipid and nonpolar drug binding (12, 13, 43, 44), is formed at the interface of chains A and B (Fig. 1*B*). The protonated, energy minimized structural model was used in unbiased molecular docking with each of the candidate Affimers as ligand. The highest probability docked complex was selected based on the balanced docking score derived from the balance coefficient. The docked complex structure was then evaluated based on the cluster number and lowest energy score. Predicted binding energies for Affimer binding based on this ClusPro docking method are shown in Table S1, the lowest energy being for Affimer A3 at −1179 kJ/mol.

**Figure 6:**
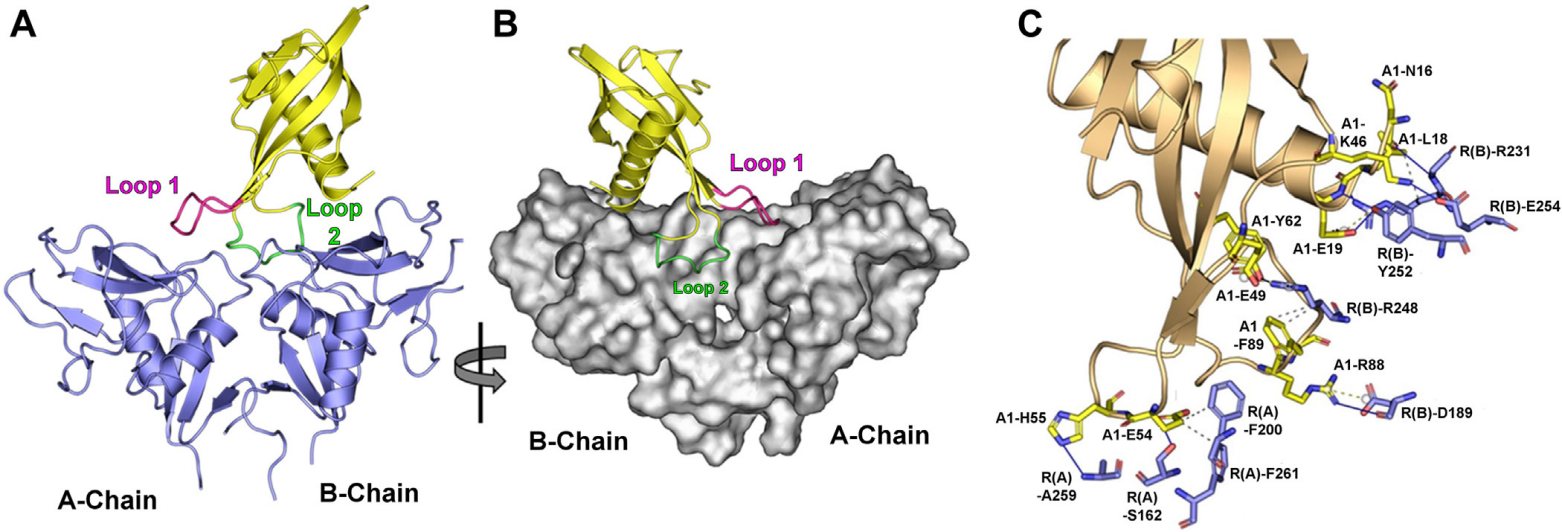
**Modeling of LOX-1/Affimer interaction.***A*, interaction of Affimer A1 (*yellow*) with LOX-1 soluble domain (*blue*). The modeling process orientates the Affimer such that variable loops VR1 and VR2 and the N-terminal end of the α-helix are in contact with uppers (oxLDL-binding) surface of the scavenger receptor. *B*, surface representation of LOX-1 with Affimer A1 as in A. The receptor is rotated 180° with respect to the orientation in A. Note the position of Affimer VR2 (Loop 2) relative to the LOX-1 “Tunnel”. *C*, predicted interactions between Affimer A1 residues (*yellow*) and LOX-1 (*Blue*: Receptor chain-A, R(A) or chain-B, R(B)). VR1 (E54 and H55,) makes interactions with chain-A, whereas interactions with chain-B are mediated *via* VR2 (R88 and F89) and N-terminal region residues L18 and E19, and non-VR residues K46, E49, and Y62. Predicted H-bonds and hydrophobic interactions are shown by *dashed* and *solid lines*, respectively. oxLDL, oxidized low-density lipoprotein; VR, variable region.

**Supplemental Fig. S2:**
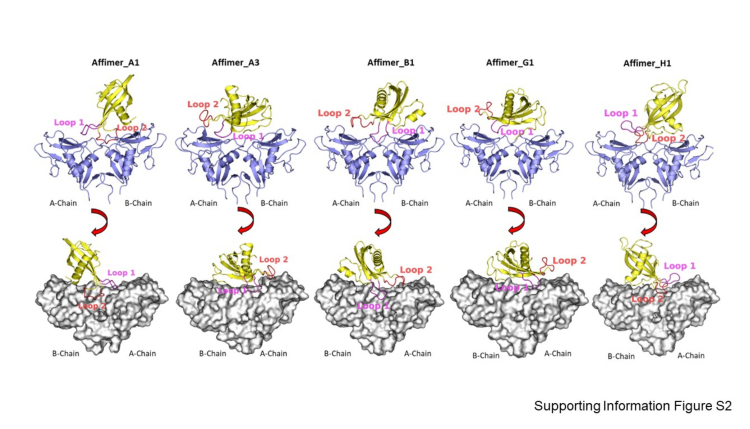
**Modeled Affimer binding to human LOX-1.** Energy-minimised models of the LOX-1-specific Affimers (*yellow ribbon* representation) bound to LOX-1 C-terminal lectin-like domain in ribbon representation (*blue*: top row) and surface representation (*grey*: bottom row). VR1 (Loop 1: magenta) and VR2 (Loop 2: *red*) are shown for each Affimer. In all cases, modeling places the Affimer with VR loops at the surface of LOX-1 that contains conserved Arg residues implicated in oxLDL binding. Views in the bottom row are rotated 180° relative to the views in the top row. Affimers A1 and H1 VR loops primarily interact with Chain-A of LOX-1 or residues close to the dimer interface, with residues around the N-terminal end of the α-helix of the Affimer associated with Chain B. For A3, B1 and G1, Affimer binding is rotated approximately 180° and tilted relative to the binding of A1 and H1 such that VR1 and VR2 and the helix N-terminal region associate almost exclusively with Chain-A. For A3, B1 and G1, C-terminal Affimer residues associate with Chain-B. For detailed predictions of interactions see main text Figure 6 and Table S2.

Models of the highest scoring sLOX-1–Affimer complexes all show Affimer binding to the surface of the CTLD domain that contains the distinctive pattern of charged and hydrophobic residues proposed to define the oxLDL binding surface (12, 13, 15, 17). For all five LOX-1-binding Affimers, interactions with the scavenger receptor were predicted to involve a range of hydrophobic interactions, H-bonds, salt bridges, π-cation, and π-stacking interactions across the putative oxLDL binding surface of LOX-1 (Table S2). Affimers A1 and H1 showed similar binding features, with the majority of H-bond and hydrophobic interactions predicted to form between Affimer variable regions and LOX-1 chain-A surface residues, with relatively few contributions to chain-A interaction from non-VR residues (Table S2; Fig. S2). Modeled interactions between these Affimers and chain-B of LOX-1 are however proposed to be predominantly with non-VR residues (Table S2). Presumably, this can occur because of favorable orientation imposed by the binding geometry of VR1/2 with chain-A. The example model of LOX-1 CTLD complexed with Affimer A1 (Fig. 6, *A*–*C*) shows the predicted pattern of interactions involving both VR1 and VR2. The principle points of contact are VR1 with the (putative) LOX-1 binding surface of chain-A and VR2 with the region of chain-B that includes Arg248. Contact between the N-terminal end of the Affimer α-helix and the region of chain-B that includes Glu254 is also predicted. This acidic residue is located at the center of the non-polar “star” pattern of residues on this surface of the receptor (as described in (13)) (Fig. 6*C*). Hence, Affimer A1 contacts both LOX-1 chains and straddles the dimer interface region, but makes no appreciable contact with (and therefore does not occlude) the “tunnel” feature at the receptor dimer interface (Fig. 6*B*)

Affimers A3, B1, and G1 show predicted binding orientations that are rotated approximately 180° around the long axis of the complex compared to A1 and H1. In all three cases, VR1 is predicted to occupy a similar position to VR2 as seen in the A1 and H1 binding models. VR2 is also predicted to occupy a similar position to VR1 in those models (Fig. S2). The majority of interactions between receptor and Affimer variable regions are therefore predicted to be with chain-A. Some non-VR interactions between chain-B and the C-terminal region of the Affimer are also predicted to contribute to binding (Fig. S2; Table S2). Overall, for all Affimers, there is considerable overlap with the LOX-1 surface regions containing the conserved arginine residues and nonpolar “star” region, both of which are implicated in oxLDL binding (12, 13, 15, 17). Glu254 on the surface of LOX-1 is not implicated in binding, but association of Affimer with this residue would clearly occlude oxLDL binding.

## Discussion

The LOX-1 scavenger receptor contributes to cardiovascular pathologies directly through its role in oxLDL uptake and accumulation, and *via* a signaling function that can be proinflammatory or proapoptotic (45, 46). Signaling *via* MAPK/ERK1/2 increases expression of adhesion molecules vascular cell adhesion molecule 1 and intracellular adhesion molecule 1 (47), and monocyte chemoattractant protein 1 (7), promoting monocyte infiltration of the blood vessel intima. This has led to significant focus on this scavenger receptor as a potential therapeutic target. Targeting strategies have included evaluating the effects of existing agents such as statins or anti-glycemics (reviewed in (48)), development of inhibitory antibodies (38) or design of new synthetic small molecule inhibitors (35, 36, 44). The effects of some natural molecules have also been evaluated (49). In this study, a novel approach was adopted: a series of small, highly stable proteins based on plant cystatin template were identified from an extensive library. These were shown to bind with high specificity to human LOX-1 extracellular domain. These Affimers also showed subnanomolar potency either as inhibitors of oxLDL binding to LOX-1, or antagonists of oxLDL-mediated signaling through MAPK (ERK1/2). Given the ongoing debate around druggability of the LOX-1/oxLDL interaction (50), discovery of a completely novel set of potential inhibitors of this interaction opens up new avenues of investigation. Affimers could act as molecular probes to interrogate the interactions between receptor and ligand, or could themselves act as template biopharmaceuticals with beneficial effects on atherosclerotic plaque formation. To date, the majority of applications for Affimers have been as probes to support high-resolution imaging (51, 52) or as antibody replacements in diagnostics and biosensor development (53, 54, 55). However, they have also proven to be powerful tools in the study of protein-protein interactions (56, 57), with the capability to discriminate between very closely related proteins (58). Affimers have also been identified that are capable of modulating biological activities, either inhibiting viral replication (59) or SUMO-mediated protein interactions (56). Affimers mimicking complement C3 that bind to fibrinogen have also been shown to support fibrinolysis, with the potential to reduce risk of thrombosis (60, 61).

A number of small molecular inhibitors have also been characterized that have specific effects on the LOX-1/oxLDL interactions. Direct comparison of these compounds with the Affimers described here is rendered difficult because of the range of assays and engineered cell lines used to evaluate them. The compound BI-0115 (36) has an IC_50_ of ∼7 μM, measured by cellular uptake of fluorescent oxLDL similar to the method used here. The best performing compound identified by Thakkar *et al.* using virtual screening and also evaluated using oxLDL uptake by LOX-1-expressing cells (35) had an IC_50_ of ∼200 nM. Compounds recently described by Tomar *et al.* (50) had *K*_D_ values measured by surface plasmon resonance in the range ∼5 to 2000 nM. Affimers achieve ≥90% inhibition of oxLDL binding at subnanomolar concentrations, with similar highly potent effects on signaling events triggered by receptor-ligand interaction. Hence, even taking into account potential problems with comparing different types of molecule using different assays, it is clear that this type of Affimer represents a very positive new contribution to the panel of potential LOX-1 inhibitors. Affimers, therefore, have significant potential to specifically target the LOX-1/oxLDL interaction *in vivo* if obstacles to biopharmaceutical development and biocompatibility could be overcome.

In general terms, computational modeling of binding of Affimers to LOX-1 shows similar outcomes in all cases (Fig. S2), with all five Affimers predicted to bind to the upper surface of the receptor. Affimers A1 and H1 are predicted to bind LOX-1 chain A *via* VR1, chain B *via* residues close to the end of the single α-helix of the Affimer, and with VR2 adjacent to the chain A-B junction. Compared to Affimers A1 and H1, Affimers A3, B1, and G1 are predicted to bind in configurations rotated around the long axis of the receptor and with varying degrees of tilt. The rigid body docking algorithm in ClusPro used in this study has limitations and cannot for example take into account likely flexibility in the Affimer VR loops. Consequently, any conformational changes induced by binding interactions are not represented in the modeling, an inherent limitation of the method used. However, the interaction modeling is not directed by any imposed constraints and is reported to have achieved consistently high confirmed reliability in predicting binding, outperforming similar docking programs for this type of application (discussed in (62)). On that basis, we can be confident that the modeling has identified the correct binding surface on LOX-1. Although the limitation of the docking algorithm in accommodating flexibility in the Affimer structure could impact on the precise geometry of binding interactions, the basic fitting is likely to be correct at low resolution. Crucially, this independently identifies the Affimer binding site on LOX-1 as being the surface containing residues known to be involved in oxLDL association. This is the same surface also identified as containing the binding sites for small molecule inhibitors of LOX-1/oxLDL interaction (36, 50).

Gaining a clear picture of the mechanism of action of the Affimers is rendered difficult by the incomplete understanding of how oxLDL interacts with the scavenger receptor. In particular, it is unclear which products of oxidation on the lipoprotein are responsible for the selectivity of LOX-1 exclusively for the oxidized form of LDL. Possibilities include (but are not limited to) modified phospholipid, fatty acids, glycation end-products, or modified or cross-linked apoprotein B100 residues leading to altered surface charge characteristics or changes in conformation. A role for glycation in binding implied by the involvement of a lectin-like domain structure is reinforced by the observations that oxLDL-dependent effects are inhibited by a polynucleoside polyinosine and by the polysaccharide carrageenan (37, 39). LDL oxidation brings about an overall increase in negative charge density, evidenced by increased rate of migration toward the anode during native gel electrophoresis. Given the distinctive distribution of positively charged residues on the surface of LOX-1, some contribution from charge interaction seems certain. In addition to the series of surface arginine residues implicated in binding (Fig. 1*B*), several other structural features of the CTLD of the receptor have been implicated in binding oxLDL. These include a number of surface “acid-base patches” comprising adjacent positively and negatively charged residues, one of which is the site of the proatherogenic K167N mutation. This mutation decreases oxLDL-stimulated ERK1/2 activation in human patient-derived macrophages (63). A third important feature of the LOX-1 lectin-like domain is the “tunnel region” formed at the dimeric interface (Fig. 1*B*) and implicated in binding of nonpolar drugs and free fatty acids (44). Small molecule inhibitors of LOX-1, presumed to bind at the “tunnel”, block oxLDL-stimulated ERK1/2 and p38 MAPK phosphorylation and activation in endothelial cells (35). However, it is unclear whether they would achieve this by preventing oxLDL binding or by uncoupling binding from conformational changes in the receptor that presumably act to transmit signal across the membrane. Alternatively, small molecular inhibitors may bind to the arginine-rich surface of LOX-1 (50), and have also been proposed to induce lectin-like domain tetramerization that occludes the oxLDL binding surface (36). Modeling of the binding of the LOX-1-binding Affimers predicts coverage only of parts of the upper surface of the lectin-like domain (the uppermost surface in Fig. 1*B*), not extending sufficiently close to either the Lys167-containing acid-base pair or the tunnel region to interfere with any contribution to oxLDL binding from these structural features. What is predicted by the modeling is that all the LOX-1-binding Affimers to some extent associate with both polypeptides of the LOX-1 dimer; hence, association is likely to occur only when a stable dimer is formed. The presence of disulfide bonds in the recombinant sLOX-1 used in the initial Affimer screen (Fig. 1*C*) indicates the presence of dimers, hence an element of consistency between the biochemical screening and modeling. Given the overall size of the Affimer library, it is perhaps not surprising that it includes five molecules with the correct spatial distribution of charged, polar, and hydrophobic residues in the VR loops to match the geometry of complementary residues across the surface of the LOX-1 dimer. As discussed above, if the modeling of Affimer binding to LOX-1 is accepted as accurate and robust, the data also provide additional evidence that it is this upper, arginine-rich surface of the receptor that is predominantly responsible for binding oxLDL. However, we cannot conclude that this surface is exclusively responsible for oxLDL binding, only that obstructing it with Affimer is sufficient to prevent stable association. Speculatively, the apparent need for stable LOX-1 dimer for Affimer binding may also contribute to the qualitative differences in plasma membrane labeling seen with anti-FLAG monoclonal antibody and fluorescently labeled Affimers (Fig. 3*B*). The Affimer could be staining only dimeric receptor in membrane subdomains linked to clathrin-independent endocytosis (40), whereas the anti-FLAG antibody may be recognizing both monomeric and dimeric protein in the over-expressed receptor population as a whole.

In conclusion, we describe a new class of synthetic proteins termed Affimers capable of targeting human LOX-1 functionality. These Affimers recognize LOX-1 either as highly purified protein or as functional, membrane-integrated receptor on the cell surface with high specificity and potency, blocking both oxLDL binding and associated intracellular signaling processes downstream of the receptor. Future work will focus on evaluating the potential of Affimers for modulation of proatherogenic or disease-associated outcomes in cell and animal models.

## Experimental procedures

### Mammalian cell culture

HEK29T cells stably expressing human LOX-1 were cultured in Dulbecco’s modified Eagle’s medium supplemented with 10% (v/v) Fetal Bovine Serum Gold (PAA Laboratories), modified Eagle’s medium nonessential amino acids, 2 mM L-glutamine, 50 U/ml penicillin and 50 μg/ml streptomycin. Cells were incubated at 37 °C in a humidified 5% CO_2_ incubator. The HEK293T cell line for tetracycline-inducible expression of human LOX-1 was engineered using the Flp-In T-Rex 293 system (Thermo Fisher Scientific). LOX-1 complementary DNA (64) was subcloned into the multiple cloning site of pcDNA5/FRT/TO eukaryotic expression plasmid which was then integrated into Flp-In T-Rex 293 cells by cotransfection with the helper plasmid. Genomic integration of pcDNA5/FRT/TO-LOX-1 was assayed using resistance to blasticidin (15 μg/ml) and hygromycin (50 μg/ml). Individual clonal populations were isolated and propagated to assay for tetracycline (1 μg/ml) inducible LOX-1-FLAG expression, detected by immunoblotting with mouse anti-FLAG M2 monoclonal antibody (Merck).

### SDS-PAGE and immunoblotting

For whole cell lysates, cells were disrupted in cell lysis buffer PBS, 2% (w/v) SDS wtih cOmplete Protease Inhibitor Cocktail (Cat. No. 0469311600, Roche Diagnostics) with a cell scraper followed by sonication on ice for 3 s using a probe sonicator. Samples were then heated at 95 °C for 5 min before determining protein concentration using the bicinchoninic acid/Cu^2+^ assay method. Typically, lysate equivalent to 25 μg total protein was separated by SDS-PAGE on 15% acrylamide gels. For immunoblotting, separated proteins were transferred to nitrocellulose. Blots were developed by enhanced chemiluminescence using Pierce ECL Western Blotting Substrate (Thermo Fisher Scientific) and images captured using the G:BOX Chemi-XT (Syngene). For protein staining after SDS-PAGE, gels were incubated with InstantBlue Protein Stain (Expedeon) and washed with distilled water.

### Recombinant LOX-1 expression and purification

Bacterial expression constructs were previously made by subcloning truncated human LOX-1 complementary DNA into pET15b vectors (64). Two constructs were made to allow the creation of different truncated soluble LOX-1 proteins: pET15b-LOX-1-ECD contains the coding region for the neck and C-type lectin-like domains preceded by N-terminal polyhistidine tag and tobacco etch virus and thrombin proteolytic cleavage sites (Fig. 1*B*). The pET15-LOX-1-CTLD is identical to this construct, but does not encode the neck region (Fig. 1*B*). Plasmids were used to transform *E.coli* BL21 DE3-Star cells and transformants selected for ampicillin resistance. For protein expression, cultures were grown from single colonies in LB culture medium with ampicillin (50 μg/ml) at 37 °C to an *A*_600_ ∼0.6. Cultures were then induced with 0.1 mM IPTG and incubated for 16 h at 25 °C for protein expression.

Cell pellets were recovered by centrifugation (4000*g*, 30 min) before resuspension in lysis buffer (10 mM Tris–HCl pH 7.8, 1 mg/ml lysozyme, 1 mM PMSF) and incubated on ice for 30 min. Cell suspensions were subjected to 6 × 30 s bursts of sonication on ice. Lysates were centrifuged at 10,000*g* for 15 min to recover LOX-1 inclusion bodies, which were then dissolved in solubilization buffer (10 mM Tris–HCl, 6 M guanidine hydrochloride (GnHCl), 100 mM sodium dihydrogen phosphate, pH 8.0). The inclusion body lysate was incubated with Ni-NTA agarose (Qiagen) for 30 min at 4 °C on a rotator wheel before packing into a chromatography column and washing with 10 to 20 column volumes of solubilization buffer supplemented with 20 mM imidazole to remove nonspecifically bound proteins. When the *A*_280_ of wash fractions was <0.05, recombinant LOX-1 proteins were eluted from the solubilization buffer containing 250 mM imidazole. Fractions containing LOX-1 were pooled and the proteins refolded as described previously (64). Refolded LOX-1 proteins were stored at −80 °C in 25% (v/v) glycerol, 25 mM Tris–HCl, 100 mM NaCl, pH7.8. Recombinant mouse LOX-1 extracellular domain was purchased from R&D Systems Inc.

### Affimer screening, isolation, and purification

Affimers against LOX-1 were isolated using a phage-display approach. The bacterially expressed soluble LOX-1 ECD variant described below was used as the target protein for selection. LOX-1 ECD was biotinylated by incubation with EZ-Link NHS-SS-Biotin (0.1 mg/ml, 60 min at 4 °C) (Thermo Fisher Scientific), which facilitated adhesion to streptavidin-coated 96-well plates (Thermo Fisher Scientific). The Affimer phage library was then incubated with the protein-bound wells to screen for LOX-1-binding Affimers as previously described (30). Coding sequences for 5 LOX-1-specific Affimers designated A1, A3, B1, G1, and H1 were isolated and cloned from the pBSTG1-Adh phagemid vector into a pET-11a-derived bacterial expression vector, including codon segments for polyhistidine tags to facilitate purification. For Affimer production, *E. coli* BL21 DE3 star cells were transformed with the pET11a plasmids corresponding to the identified anti-LOX-1 Affimers A1, A3, B1, G1, or H1. The same approach was used to generate a negative control Affimer, 37C. Affimer expression and purification was performed essentially as described above, with the exception that lysis buffer contained 1% w/v Triton X-100 instead of GnHCl because expressed Affimers are fully folded and soluble.

### Affimer labeling

The Affimer C-terminal cysteine residue can be modified *via* maleimide chemistry. Each Affimer (0.5 mg/ml) was incubated with tris-(2-carboxyethyl) phosphine immobilized resin (Thermo Fisher Scientific) for 1 h at room temperature. The tris-(2-carboxyethyl) phosphine resin was then pelleted by centrifugation (1500*g* for 1 min), and the supernatant was transferred to a fresh tube. Affimer was then modified with 0.1 mM biotin-maleimide or AlexaFluor 488 maleimide (Merck) by incubation for 2 h at room temperature. Unbound maleimide reagents were removed using a Zeba 7 kDa MWCO centrifugal desalting column (Thermo Fisher Scientific). Biotinylation was assayed by separation on SDS-PAGE and electrophoretic transfer to nitrocellulose membrane before blocking with 0.1% (w/v) BSA and probing with High Sensitivity Streptavidin-horseradish peroxidase (HRP) (Thermo Fisher Scientific) at 1:10,000 dilution in phosphate-buffered saline with 0.05% Tween-20 (PBST) for 1 h at room temperature. Blots were imaged as described above. Fluorophore conjugation was assayed by fluorescence imaging *in situ* after SDS-PAGE using the G:Box ChemiXT *via* fluorescence excitation at 488 nm and capturing emission at 520 nm.

### ELISA assay of sLOX-1/Affimer interaction

Triplicate samples of sLOX-1-ECD, sLOX-1-CTLD, recombinant mouse sLOX-1 (Bio-Techne, Cat. No:1564-LX), or BSA (Thermo Fisher Scientific) suspended in ELISA Buffer A (2.5 mM NaH_2_PO_4_, 7.5 mM Na_2_HPO_4_, 145 mM NaCl, pH 7.2) were added to the wells of a Maxisorp 96-well plate (Nunc) and incubated overnight at 4 °C. Plates were washed 3× with ELISA buffer B (2.5 mM NaH_2_PO_4_.2H_2_O, 7.5 mM Na_2_HPO_4_, 500 mM NaCl, 0.2% (w/v) Tween-20, pH 7.2). To reduce nonspecific binding, 100 μl of 0.1% (w/v) BSA in ELISA buffer A was added to wells and incubated for 1 h at 4 °C. Plates were again washed 3× with buffer B. Biotinylated Affimers (1 μg/ml in buffer B) were added and the plate incubated on a rocking platform for 2 h at room temperature. Plates were again washed 3× with buffer B before addition of streptavidin-HRP (1:16,000 dilution in buffer B) before being incubated for 1 h at room temperature. Plates were again washed 3× with buffer B before Affimer binding was quantified from color development with *o*-phenylenediamine/sulphuric acid solution. Absorbance at 492 nm was measured using a Varioskan Flash Microplate reader (Thermo Fisher Scientific).

### LDL and oxLDL preparation

Blood was taken from consenting volunteers in accordance with local ethical approval and license (University of Leeds #BIOSCI 15–007), with 0.38% (w/v) trisodium citrate to prevent coagulation. Plasma was isolated by centrifugation (3000*g*, 20 min) and mixed with OptiPrep density gradient medium at a 4:1 ratio and subjected to ultracentrifugation at 600,000*g* for 3 h at 16 °C. The orange/yellow LDL band was removed, dialyzed against PBS and adjusted to 1 mg/ml protein for storage at 4 °C in the dark. The LDL was oxidized by incubation with 5 μM CuSO_4_ for 24 h at 37 °C in the dark before addition of EDTA (100 μM) and butylated hydroxytoluene (20 μM) to stop further oxidation. Oxidation was assessed by mass shift during agarose gel electrophoresis as described (65). oxLDL was labeled with the lipophilic dye Dil (1,1′-dioctadecyl-3,3,3′,3′-tetramethylindodicarbocyanine-5,5′-disulfonic acid; Thermo Fisher Scientific) at a ratio of 0.3 mg dye (in dimethyl sulfoxide) per mg of lipoprotein particles by incubation at 37 °C for 18 h. Samples were then dialyzed against two changes of PBS over a 24 h period to remove dimethyl sulfoxide and unincorporated Dil. Labeled lipid particles were collected from dialysis and stored at 4 °C, protected from light. Successful labeling was confirmed by agarose gel electrophoresis followed by fluorescence imaging on the G-BOX Chemi-XT with 550 nm excitation and 570 nm emission.

### Assay of oxLDL binding to HEK293T-LOX-1 cells

HEK293-LOX-1-FLAG cells were seeded onto poly-L-lysine coated glass coverslips at 25% confluence. Upon reaching 50% confluence, cells were induced to express LOX-1-FLAG by changing the culture medium to OptiMEM, 0.2% BSA ± tetracycline (1 μg/ml). After 16 h, cells were incubated on ice for 30 min with fresh medium ± Affimer at 0.01, 0.1 or 1.0 μg/ml or the anti-LOX-1 antibody JTX92 (1 μg/ml). Dil-labeled oxLDL (10 μg/ml protein) was then added and the incubation continued for 30 min. Cells were washed 3× with PBS (with Ca^2+^/Mg^2+^) before fixation with 3% (w/v) paraformaldehyde for 15 min. Cells were further washed 3× with PBS before incubation with 4′,6-diamidino-2-phenylindole (DAPI) (1 μg/ml) for 30 min at room temperature. After further washing with PBS, coverslips were mounted in Fluoromount G onto glass slides. Images were captured with a Zeiss LSM700 confocal microscope using the Plan-Apochromat 40×/1.30 oil objective lens. DAPI was excited at 405 nm (emission at 400–500 nm) and Dil-labeled oxLDL was excited with at 555 nm with emission monitored at 610 nm. Dil-labeled oxLDL was quantified from the microscopy images using ImageJ (66). For fluorescence microscopy analysis of binding of fluorescently labeled affimers, HEK293T-LOX-1 cells were seeded onto coverslips, induced, and grown as above. After 16 h induction, the culture medium was replaced with OptiMEM containing AlexaFluor488-labeled Affimers (25 μg/ml), with incubation on ice for 30 min. Coverslips were washed, fixed, stained with DAPI, and mounted for microscopy as above. For staining of LOX-1-FLAG, cells were fixed with paraformaldehyde as above and incubated with a 1:5000 dilution of mouse anti-FLAG M2 antibody (Cat. No. F1804; Proteintech) in PBS with 0.5% (w/v) BSA for 1 h at room temperature. After 3 × 20 min washes with PBS, the coverslips were incubated with donkey anti-mouse AlexaFluor 488 conjugate (Cat. No. A32766; Thermo Fisher Scientific) in PBS with 0.1% (w/v) BSA and DAPI (1 μg/ml). After 2 h incubation at room temperature and 3 × 20 min washes with PBS, the coverslips were mounted and imaged as above.

### Assay of ERK1/2 activation

HEK293T-LOX-1 cells were cultured in 6-well plates to 50% confluency before being induced to express LOX-1-FLAG as described above. After 16 h, the medium was replaced with fresh OptiMEM, supplemented with Affimer A1 or H1 (10 or 100 ng/ml) where appropriate, and incubated at 37 °C for 30 min. Cells were incubated with oxLDL (10 or 100 μg/ml) for varying times to stimulate signaling *via* LOX-1. Medium was aspirated from the plate and the wells washed 3× with PBS before extraction into cell lysis buffer and processing for SDS-PAGE, as above. A similar approach was used to detect direct stimulation of ERK1/2 activation by Affimers acting as agonist, but without addition of oxLDL and starting the time course at the point of Affimer addition to the cells. Subsequent immunoblots were probed with the following antibodies: Rabbit anti-ERK1/2 (Cat. No. 9102), rabbit anti-phospho-ERK1/2 (Cat. No. 9101) all from Cell Signaling Technology, mouse anti-α-tubulin (Cat. No. 66031; Proteintech) before development with goat anti-rabbit HRP conjugate (Cat. No. 111–035–144; Jackson ImmunoResearch). Immunoblots were developed and chemiluminescence captured as described above. Immunoblot signal was quantified using ImageJ by measuring the mean integrated density of bands and subtracting the background signal captured within the same lane.

### Modeling of sLOX-1 and Affimer interaction

Amino acid sequences for the five LOX-1-interacting Affimers were aligned using ESPript/ENDscript, (67). Individual Affimers were modeled using I-TASSER (Iterative Threading ASSEmbly Refinement) tools (68). I-TASSER uses a hierarchical approach to protein structure prediction and structure-based function annotation, constructing models based on multiple threading approach. Affimer models were selected based on C-Score, a confidence measure based on the significance of the threading template alignments and the convergence parameters of the structure assembly simulations. The 3-D structure of LOX-1 soluble extracellular domain was downloaded from the NCBI “Structure” database (PDB code: 6Tl9 (36)) from Protein Data Bank. This crystal structure is a homotetramer with 2.7 Å resolution. Models were energy-minimized using UCSF Chimera (69). Protein-protein docking was performed using the online docking server ClusPro2.0 (62). ClusPro applies rigid body methods in three computational steps: (i) rigid body docking using the fast Fourier transform correlation approach, (ii) RMSD-based clustering of the models generated to find the largest cluster that will represent the most likely structure of the complex, and (iii) refinement of the selected structure by CHARMM minimization. The highest likelihood docked complex was selected based on the Balanced Docking Score, derived from the balance coefficient. Surface electrostatics of LOX-1 extracellular domain with and without bound Affimer were evaluated using the Adaptive Poisson-Boltzmann solver (APBS 2.1; https://wiki.pymol.org/index.php/Apbsplugin) plug-in tool within PyMOL. Electrostatic potential is displayed as a color-coded electrostatic surface (units k_B_T/e).

### Statistical analysis

Two-way analysis of variance (ANOVA) followed by Dunnet’s or Tukey’s post hoc multiple comparisons tests was performed using GraphPad Prism software (https://www.graphpad.com/). Significant differences between control and test groups were evaluated with *p* values <0.05 (∗) or <0.01 (∗∗).

## Data availability

Data sets contributing to this study are archived by the corresponding author, Dr S. Ponnambalam, University of Leeds, and are available on request.

## Supporting information

This article contains supporting information.

## Conflicts of interest

M. J. M. and D. C. T. are named inventors of the Affimer technology and this is filed under U.S. patent number #10,844,370 assigned to the University of Leeds on “Scaffold proteins derived from plant cystatins”.
