# Supplementary material for: “Affimer” synthetic protein scaffolds block oxidized LDL binding to the LOX-1 scavenger receptor and inhibit ERK1/2 activation
Source: J Biol Chem. 2023 Oct 5;299(11):105325. doi: 10.1016/j.jbc.2023.105325 (PMC10641530; doi:10.1016/j.jbc.2023.105325)
Supplement: Supplemental Table S1 [file mmc1.docx]

| **Supporting Information Table S1. Parameters for modeling Affimer-LOX-1 binding** | | |
| --- | --- | --- |
| **Affimer** | **C-score** | **Weighted Score (Lowest Energy)** |
| Affimer A1 | -0.83 | -830.0 |
| Affimer A3 | -0.26 | -1179.1 |
| Affimer B1 | -0.73 | -1052.8 |
| Affimer G1 | -0.83 | -890.9 |
| Affimer H1 | -0.66 | -890.6 |
