# Supplementary material for: “Affimer” synthetic protein scaffolds block oxidized LDL binding to the LOX-1 scavenger receptor and inhibit ERK1/2 activation
Source: J Biol Chem. 2023 Oct 5;299(11):105325. doi: 10.1016/j.jbc.2023.105325 (PMC10641530; doi:10.1016/j.jbc.2023.105325)
Supplement: Supplemental Table S2 [file mmc2.docx]

| **Supporting Information S2. Modelled interactions between Affimers and LOX-1** | | | | | |
| --- | --- | --- | --- | --- | --- |
| **Hydrophobic Interactions** | | | **Hydrogen Bonds** | | **Other** |
| **Chain A - Affimer** | **Chain B - Affimer** | | **Chain A - Affimer** | **Chain B - Affimer** | **LOX-1 A/B - Affimer** |
| **Affimer A1** | | | | | |
| **F200 - E54 (3.78Å)**  **F261 - E54 (3.77Å)** | | R231 - 18L (3.98 Å)  **R248 - F89 (3.76 Å)** | **S162 - E54 (2.84 Å)**  **A259 - H55 (3.37)** | **D189 - R88 (3.95 Å)**  R231 - L18 (2.94 Å)  G232 - N16 (3.66 Å)  R248 - Y62 (2.91 Å)  Y252 - E19 (2.56 Å) E254 - K46 (2.62 Å) | **Salt Bridges**  **D189 (B) - R88 (3.35 Å)**  R231 (B) - E19 (2.97 Å)  R248 (B) - E49 (3.68 Å) |
| **Affimer_A3** | | | | | |
| **F200 - P53 (3.89 Å)**  **F200 - N54 (3.77 Å)**  **P201 - W57 (3.63 Å)**  **W203 - W57 (3.70 Å)**  **Y238 - W92 (3.96 Å)**  **Y245 - I93 (3.86 Å)**  Q247 - W82 (3.40 Å)  **Y252 - I93 (3.71 Å)**  **L258 - W92 (3.59 Å)**  **L258 - W57 (3.41 Å)**  **A259 - A55 (3.86 Å) A260 - W57 (3.47 Å)** | | L258 - A111 (3.72 Å)  L258 - H113 (3.65 Å)  F261 - H118 (3.90 Å) | **S162 - 54N (2.70 Å)**  S196 - 118H (2.83 Å)  **A233 - 92W (4.10 Å)**  **Q247 - 59Q (3.19 Å)**  R248 - 117H (2.84 Å)  **E254 - 93I (3.19 Å)**  **A259 - 54N (2.85 Å)** | S162 - H116 (2.82 Å)  S165 - H118 (4.03 Å)  Q247 - H114 (3.31 Å)  R248 - H116 (3.13 Å)  E254 - H113 (2.92 Å)  A259 - H115 (3.13 Å | **Salt Bridges**  R231 (A) - E97 (3.10 Å) |
| **Affimer_B1** | | | | | |
| **F200 - L57 (3.58Å)**  **A259 - W86 (3.60Å)**  **F261 - L57 (3.77Å)** | | F200 - W82 (3.44Å)  P201 - H119 (3.75Å)  A233 - H113 (3.93Å) | S199 - K84 (3.87Å)  **E154 - K91 (2.73Å)**  **A259 - N90 (2.78Å)** | S162 - H119 (2.89Å)  **S196 - E55 (3.99Å)**  **S199 - D59 (2.53Å)**  S199 - K84 (2.83Å)  Q236 - H114 (2.89Å)  Y238 - H114 (4.05Å)  Y245 - H117 (3.24Å)  Q247 - H119 (3.94Å)  R248 - Q50 (3.82Å)  **R248 - 51T (3.10Å)**  E254 – H113 (2.76Å) | **Salt Bridges**  R248 (B) – D59 (3.22 Å)  **π-Stacking**  Y245 (B) - H117 |
| **Affimer_G1** | | | | | |
| **P201 - L56 (3.77 Å)**  Q247 - W82 (3.70 Å)  Y252 - F95 (3.59 Å)  **L158 - L56 (3.88 Å)**  **A260 - L56 (3.87 Å)** | | L258 – H114 (3.71 Å) | **S199 - Q52 (2.75Å)**  R248 - H119 (2.77Å)  Y252 - E97 (2.60Å)  E254 - K84 (2.72Å)  **A259 - L56 (2.94Å)** | S162 - H118 (2.89Å)  S199 - Q50 (4.08Å)  R248 - G7 (2.70Å)  R248 - Q50 (2.83Å) | **Salt Bridges**  R231 (A) - E97 (3.10 Å)  **π-Stacking**  Y245 (B) - H114  **π-Cation Interactions**  R248(A) - H119 (4.41 Å)  R248 (B) - H116 (5.35 Å) |
| **Affimer_H1** | | | | | |
| **F200 - I88 (3.49Å)**  **P201 - W86 (3.75Å)**  **Y245 - W86 (3.89Å)**  **F261 - L91 (3.94Å)** | | F200 – A5 (3.99Å)  L258 –P12 (3.90Å)  A259 –P12 (3.58Å) | **S162 - S89 (2.66Å)**  S199 – T60 (3.68Å)  **R231 - H53 (2.76Å)**  **G232 - D55 (2.91Å)**  **Q247 - W86 (3.90Å)**  R248 - A5 (2.86Å)  **Y252 - L54 (3.93Å)**  **A259 - S89 (2.96Å)** | A233 - N16 (2.87Å)  Y245 - N14 (3.61Å)  Q247 - N14 (2.99Å)  Q247 - E19 (2.94Å)  R248 - N94 (2.80Å)  E254 - N16 (3.00Å)  E254 – E15 (3.16Å)  E254 - N16 (2.84Å) | **Salt Bridges**  **R229 (A) - D55 (3.88Å)** |
